# Rapid Identification of Carbapenemase Genes in Gram-Negative Bacteria with an Oligonucleotide Microarray-Based Assay

**DOI:** 10.1371/journal.pone.0102232

**Published:** 2014-07-28

**Authors:** Sascha D. Braun, Stefan Monecke, Alexander Thürmer, Antje Ruppelt, Oliwia Makarewicz, Mathias Pletz, Annett Reißig, Peter Slickers, Ralf Ehricht

**Affiliations:** 1 Alere Technologies GmbH, Jena, Germany; 2 Technische Universität Dresden, Medizinische Fakultät “Carl Gustav Carus”, Dresden, Germany; 3 Center for Infectious Diseases and Infection Control and Center for Sepsis Care and Control, Jena University Hospital, Jena, Germany; 4 Center for Sepsis Care and Control, Jena University Hospital, Jena, Germany; Amphia Ziekenhuis, Netherlands

## Abstract

Rapid molecular identification of carbapenemase genes in Gram-negative bacteria is crucial for infection control and prevention, surveillance and for epidemiological purposes. Furthermore, it may have a significant impact upon determining the appropriate initial treatment and greatly benefit for critically ill patients. A novel oligonucleotide microarray-based assay was developed to simultaneously detect genes encoding clinically important carbapenemases as well as selected extended (ESBL) and narrow spectrum (NSBL) beta-lactamases directly from clonal culture material within few hours. Additionally, a panel of species specific markers was included to identify *Escherichia coli*, *Pseudomonas aeruginosa*, *Citrobacter freundii/braakii*, *Klebsiella pneumoniae* and *Acinetobacter baumannii*. The assay was tested using a panel of 117 isolates collected from urinary, blood and stool samples. For these isolates, phenotypic identifications and susceptibility tests were available. An independent detection of carbapenemase, ESBL and NSBL genes was carried out by various external reference laboratories using PCR methods. In direct comparison, the microarray correctly identified 98.2% of the covered carbapenemase genes. This included *bla*VIM (13 out of 13), *bla*GIM (2/2), *bla*KPC (27/27), *bla*NDM (5/5), *bla*IMP-2/4/7/8/13/14/15/16/31 (10/10), *bla*OXA-23 (12/13), *bla*OXA-40-group (7/7), *bla*OXA-48-group (32/33), *bla*OXA-51 (1/1) and *bla*OXA-58 (1/1). Furthermore, the test correctly identified additional beta-lactamases [*bla*OXA-1 (16/16), *bla*OXA-2 (4/4), *bla*OXA-9 (33/33), OXA-10 (3/3), *bla*OXA-51 (25/25), *bla*OXA-58 (2/2), CTX-M1/M15 (17/17) and *bla*VIM (1/1)]. In direct comparison to phenotypical identification obtained by VITEK or MALDI-TOF systems, 114 of 117 (97.4%) isolates, including *Acinetobacter baumannii* (28/28), *Enterobacter spec*. (5/5), *Escherichia coli* (4/4), *Klebsiella pneumoniae* (62/63), *Klebsiella oxytoca* (0/2), *Pseudomonas aeruginosa* (12/12), *Citrobacter freundii* (1/1) and *Citrobacter braakii* (2/2), were correctly identified by a panel of species specific probes. This assay might be easily extended, adapted and transferred to point of care platforms enabling fast surveillance, rapid detection and appropriate early treatment of infections caused by multiresistant Gram-negative bacteria.

## Introduction

Carbapenems are last-line antimicrobial substances with a broad spectrum and high efficacy. Originally, they were developed from thienamycin, a substance that is produced by *Streptomyces cattleya* [1]. As all beta-lactam antibiotics, carbapenems inhibit the D-alanyl-D-alanine carboxypeptidase and therefore they interfere with the cell wall synthesis [2]. They are stable against penicillin and cephalosporin hydrolyzing enzymes that are commonly encountered in human pathogens. In Western Europe, four different carbapenems are available so far: imipenem, meropenem, ertapenem and doripenem. These antibiotics are exclusively used in the clinical environment as they can be administered only intravenously.

Carbapenems are recommended by major guidelines as empiric treatment for critically ill patients at risk for multi-drug resistant pathogens as well as for the therapy of surgical and intra-abdominal infections, severe urinary tract and renal infections, nosocomial pneumonia and sepsis [3]–[5]. Due to their broad spectrum, they play an important role in the treatment of mixed infections as frequently encountered in abdominal and trauma surgery, as well as for the presumptive or calculated therapy of infections as long as identification and susceptibility test results are pending. Carbapenems are considered the major treatment option for infections caused by Gram-negative bacteria that produce extended spectrum beta-lactamases (ESBL) conferring resistance against 3^rd^ generation cephalosporins. During recent years, a worldwide rise of ESBL producing bacteria in clinical settings has resulted in an increasing dependence on carbapenem compounds, especially in surgical and intensive care units [6]. The increasingly frequent application of carbapenems induces a selective pressure on bacteria to acquire resistance against carbapenems.

Antimicrobial resistance against carbapenems in *Enterobacteriacae*, and occasionally also in other Gram-negative bacteria can be caused by high level expression of ESBLs accompanied by changed porin expression [7], [8], or by specific enzymes named carbapenemases. These enzymes hydrolyze carbapenems, but also most, or all, other beta-lactam antibiotics. Several carbapenemases have been known already for more than 20 years (i.e., *bla*IMP, *bla*IMI) but have been restricted to certain species and geographic regions. For instance plasmid-mediated *IMP*-type carbapenemases first appeared in Japan in the 1990s, where they were described in *Pseudomonas* and *Acinetobacter* species [9]. Recently, different carbapenem hydrolyzing enzymes were described, out of which *bla*KPC [10], [11], *bla*NDM [12]–[14], *bla*VIM [15], *bla*GIM [16], [17], *bla*OXA-23 [18], [19] and *bla*OXA-48 [10] can frequently be found in clinical bacterial isolates. There are regional differences affecting the overall prevalence of carbapenem resistance as well as the types of carbapenemases most likely to be encountered. The prevalence of carbapenem resistance in Germany in 2011 and 2012 was comparable low (less than 1% of clinical *Klebsiella* isolates [20]). The most common carbapenemase types were *bla*OXA-23 and *bla*OXA-48 accounting for 47% of all identified carbapenemase genes [21]. In contrast, in Greece, nearly 60% of clinical *Klebsiella* isolates are resistant to carbapenems [22] and resistance is almost exclusively caused by *bla*KPC type carbapenemases [10]. Other carbapenem hydrolyzing enzymes like *bla*DIM, *bla*SME, *bla*IMI, *bla*NMC, *bla*CcrA and *bla*CMY are comparably rare and rather restricted in both their geographic incidence and in the range of bacteria in which they can be detected. Relevant resistance genes are sometimes localized on plasmids that additionally harbor toxin-antitoxin (TA) systems [23], [24]. These are also known as postsegregational cell killing and addiction systems, and essentially prevent the loss of plasmids during cell divisions [25], [26], even in the absence of external selective pressure. Therefore, they prevent the loss of a plasmid with resistance genes even after discontinuation of a therapy. This might contribute to the self-sustained presence of resistance genes in bacterial populations outside healthcare settings.

A phenotypic, presumptive detection of carbapenemases is usually performed by automated systems, like VITEK 2 (bioMérieux) or Phoenix (BD), or manually by agar-diffusion or agar-dilution assays using one carbapenem or several compounds. However, the interpretation of uncommon resistance pattern is often complicated by masking effects yielding false-positive or false negative results [27], [28] that can lead to inappropriate antibiotic treatment and therapy failure [29]. Beside proper species identification, several tests are performed in case of resistance for further confirmation. Phenotypic tests include the modified Hodge-test [30] that excludes the possibility of carbapenem resistance due to porin mutations [7], [8] by establishing the presence of a hydrolyzing enzyme secreted into the growth medium and the detection of synergy with EDTA to identify carbapenemases dependent on the presence of metal ions. Phenotypic tests generally need time and experienced staff and, furthermore, do not allow a detailed molecular identification of a carbapenemase which is needed for epidemiological and infection control purposes. Therefore, molecular methods are additionally required for an exact, fast and sensitive determination of carbapenemase genes. Recently, several PCRs and real-time PCRs have been established [31]–[33]. However, because of the high number of different target genes, several tests must be performed. This needs time, resources and manpower and therefore, common carbapenemases will be targeted only in a first series of tests. The approach may hence cause a considerable delay to obtain identification also for rare carbapenemase genes. This could be solved by combining all relevant targets in a single test using a microarray for detection and identification of multiplex PCR products generated from suspect clonal culture material [34], [35]. Such an approach could allow one to distinguish all relevant carbapenemase genes in a single experiment. For this purpose, an oligonucleotide microarray was designed, established and tested that can simultaneously analyze the most important carbapenemase genes. Using a similar technology as previously described for many different genetic marker genes in Gram-negative and Gram-positive bacteria [36], we aimed to develop a high-throughput, cost-effective assay to detect carbapenemase genes in different species of Gram-negative bacteria.

## Materials and Methods

### Bacterial strains, growth conditions, and genomic DNA extraction

The described assay was verified using a set of 117 phenotypically and genotypically characterized reference strains and clinical isolates (Table S1) from different sources [University Medical Center of Dresden (n = 88), University Medical Center of Jena (n = 14), German Collection of Microorganisms and Cell Cultures (DSMZ; n = 3), Institut Pasteur Paris (n = 1), Friedrich-Loeffler-Institute Jena (n = 2) and the German National Reference Laboratory for Multidrug-Resistance Gram-negative Bacteria at Bochum University (NRL, n = 9)]. The investigated strain collection comprised *Citrobacter freundii* (n = 1), *Citrobacter braakii* (n = 2), *Escherichia coli* (n = 4), *Enterobacter cloacae* (n = 5), *Klebsiella oxytoca* (n = 2), *Klebsiella pneumoniae* (n = 63), *Acinetobacter baumannii* (n = 28), *Pseudomonas aeruginosa* (n = 12). All strains and isolates were tested for resistance to carbapenems using automated microdilution techniques for meropenem and imipenem (VITEK 2, bioMérieux, Nuertlingen, Germany) and agar diffusion assays with meropenem and ertapenem disks according to EUCAST guidelines [37], or e-tests (bioMérieux). All phenotypically carbapenem-resistant isolates were additionally tested with a modified Hodge-Test [30] to identify isolates with AmpC β-lactamases in combination with porin loss [7], [8]. Isolates were cultivated on tryptone yeast agar. Genomic DNA was extracted using the Roche High Pure PCR Template Preparation Kit (Roche Diagnostics, Germany) or the Qiagen DNeasy Blood & Tissue Kit (Qiagen GmbH, Hilden, Germany) according to manufacturer's instructions after treatment with lysis enhancer (Alere Technologies, Jena, Germany). If necessary, DNA was concentrated to at least 100 ng/µl using a SpeedVac centrifuge (Eppendorf, Hamburg, Germany) at room temperature for 30 min/1,400 rpm. In order to further reduce costs and to save time, a fast and robust heat lysis protocol was developed. 50 µl molecular grade water were used to homogenize a loop full (1 µl loop size, e.g. VWR order no. 612–9355) of fresh clonal bacterial culture directly harvested from a TY agar plate. The mixture was heated in a safe lock Eppendorf tube at 99°C in an Eppendorf Thermoshaker (Eppendorf) for 15 min. After centrifugation (5 min, 16,000 g), 25 µl of the supernatant were subsequently treated by RNase A (5 min, 37°C) at a final concentration of 10 µg/ml. 5 µl of recovered genomic DNA were used directly for internal biotin-labeling and subsequently for hybridization.

### Primer and probe design

Hybridization probes and labeling primer were designed to discriminate the given set of relevant carbapenemases as well as genes characteristically found only in certain taxa of *Enterobacteriales* and *Pseudomonadales* (Table 1, Tables S2 and S3). The later allow the determination of the genus or species of the bacterial colony under investigation. For each target gene all sequences available in GenBank (http://www.ncbi.nlm.nih.gov/nuccore/) were downloaded and used to construct a multi sequence alignment. In most cases the multi sequence alignment comprises several allelic or SNP variants. The probe and primer binding sites were preferentially placed into the most conserved regions of the coding sequence. For most target genes more than one none overlapping probe/primer set was designed to prove their performance in an experimental comparison. Length of primers (16–27 bp) and probes (22–32 bp) were varied in order have uniform binding affinities for all oligonucleotides. The nearest neighbor parameter set published by SantaLucia in 1998 [38] was utilized to calculate Tm values. Carbapenemase probes (n = 42) and their corresponding primers were designed by analyzing all available and correctly annotated GenBank sequences related to the genes *bla*KPC, *bla*VIM, *bla*IMP, *bla*GIM, *bla*IND, *bla*NDM, *bla*KHM, *bla*OXA-23, *bla*OXA-54, *bla*OXA-55, *bla*OXA-58 and genes related to the *bla*OXA-40-, *bla*OXA-48- and *bla*OXA-51-group. Additionally, probes (n = 25) specifying clinically important NSBLs and ESBLs *bla*OXA-1, *bla*OXA-2, *bla*OXA-7, *bla*OXA-9, *bla*OXA-10, *bla*OXA-60 and *bla*CTX-M1 (Table 1 and Tables S2, S3 and S4) were incorporated. Furthermore, primer-probe combinations (n = 15) for species identification were included for *Acinetobacter baumannii* (*basC, efp, pld*), *Klebsiella pneumoniae* (*khe*), *Pseudomonas aeruginosa* (*ecfX*), *Citrobacter freundii*/*braakii* (*cfa*) and *Escherichia coli* (*gad*) and for the genus *Enterobacter* a combination of *dnaE* and *ihfA* were included (Table 1 and Tables S2, S3 and S4).

**Table 1:** Target overview of the microarray-based carbapenemases assay.

Probe Function	Target	Gene Function	Reference Sequence
Species-specific marker	*basC*	acinetobactin biosynthesis of *Acinetobacter baumannii*	AY571146.1 [6565∶7875]
Species-specific marker	*cfa*	colicin biosynthesis of *Citrobacter freundii*/*braakii*	U09771.1 [1∶271]
Species-specific marker	*dnaE*	alpha subunit DNA polymerase III of *Escherichia coli*	U00096.2 [205126∶208608]
Species-specific marker	*ecfX*	extracellular sigma factor of *Pseudomonas aeruginosa*	DQ996558.1 [1∶528]
Species-specific marker	*efp*	elongation factor P of *Acinetobacter baumannii*	CP001172.1 [1124029∶1124598]
Species-specific marker	*gad*	glutamate decarboxylase of *Escherichia coli*	AE014075.1 [1756318∶1757787]
Species-specific marker	*ihfA*	alpha subunit of the integration host factors of *Escherichia coli*	U00096.2 [1793277∶1793576]
Species-specific marker	*khe*	klebsolysine of *Klebsiella pneumoniae*	AF293352.1 [91∶579]
Species-specific marker	*pld*	phospholipase D of *Acinetobacter baumannii*	CP000521.1 [3477433∶3479057]
Beta lactamase	*bla*OXA-1	narrow spectrum beta lactamase, class D beta-lactamase	AY458016.1 [10718∶11548]
Beta lactamase	*bla*OXA-2	narrow spectrum beta lactamase, class D beta-lactamase (Synonym: blaOXA 53 in *Salmonella enterica*)	Consensus (OXA-2-group)
Beta lactamase	*bla*OXA-7	narrow spectrum beta lactamase, class D beta-lactamase	AY866525.1 [1931∶2731]
Beta lactamase	*bla*OXA-9	narrow spectrum beta lactamase, class D beta-lactamase	M55547.1 [2314∶3198]
Beta lactamase	*bla*OXA-10-group	narrow spectrum beta lactamase, class D beta-lactamase	Consensus (OXA-10-group)
Beta lactamase	*bla*OXA-60	narrow spectrum beta lactamase, class D beta-lactamase	AF525303.2 [2771∶3586]
Beta lactamase	*bla*CTX-M1/M15	extended-spectrum-beta-lactamase, class A beta-lactamase	X92506.1 [63∶938]
Carbapenemase	*bla*OXA-23	carbapenemase, class D beta-lactamase	AJ132105.1 [972∶1793]
Carbapenemase	*bla*OXA-40-group	carbapenemase, class D beta-lactamase	Consensus (OXA-40-group)
Carbapenemase	*bla*OXA-48-group	carbapenemase, class D beta-lactamase	Consensus (OXA-48-group)
Carbapenemase	*bla*OXA-51-group	carbapenemase, class D beta-lactamase	Consensus (OXA-51-group)
Carbapenemase	*bla*OXA-54	carbapenemase, class D beta-lactamase	AY500137.1 [1∶798]
Carbapenemase	*bla*OXA-55	carbapenemase, class D beta-lactamase	AY343493.1 [77∶946]
Carbapenemase	*bla*OXA-58	carbapenemase, class D beta-lactamase	AY665723.1 [3301∶4143]
Carbapenemase	*bla*GIM	carbapenemase, class B metallo beta-lactamase	AJ620678.1 [1041∶1793]
Carbapenemase	*bla*IND	carbapenemase, class B metallo beta-lactamase	Consensus
Carbapenemase	*bla*KHM	carbapenemase, class B metallo beta-lactamase	AB364006.1 [62∶787]
Carbapenemase	*bla*KPC	carbapenemase, class B metallo beta-lactamase	EU447304.1 [15∶896]
Carbapenemase	*bla*NDM	carbapenemase, class B metallo beta-lactamase	FN396876.1 [2407∶3219]
Carbapenemase	*bla*IMP	carbapenemase, class B metallo beta-lactamase	Consensus
Carbapenemase	*bla*VIM	carbapenemase, class B metallo beta-lactamase	Consensus
Carbapenemase	*bla*VIM-2	carbapenemase, class B metallo beta-lactamase	AF191564.1 [1316∶2116]
Carbapenemase	*bla*VIM-7	carbapenemase, class B metallo beta-lactamase	AJ536835.1 [150∶947]

**Table 2:** Carbapenemases genotyping results of the **M**icroarray-based assay in comparison to the genotyping results of carbapenemases genes obtained from different reference laboratories that used standard PCR as **R**eference method.

Species^a^	No. of isolates	No^b^. with *bla*KPC gene		% Concordance of *bla*KPC results	No^c^. with *bla*VIM gene		% Concordance of *bla*VIM results	No^d^. with *bla*NDM gene		% Concordance of *bla*NDM results	No^e^. with *bla*GIM gene		% Concordance of *bla*GIM results	No^f^. with *bla*OXA-48-like gene		% Concordance of *bla*OXA-48-like results	No^g^. with *bla*OXA-23 gene		% Concordance of *bla*OXA-23 results	No^h^. with *bla*OXA-58 gene		% Concordance of *bla*OXA-58 results	No^i^. with *bla*OXA-40-like gene		% Concordance of *bla*OXA-40-like results	No^j^. with *bla*OXA-51-like gene		% Concordance of *bla*OXA-51-like results	No^k^. with *bla*IMP gene		% Concordance of *bla*IMP results	No. of strains that agree/total no. of strains	% Concordance of all strains
		R^l^	M^m^		R	M		R	M		R	M		R	M		R	M		R	M		R	M		R	M		R	M			
*A. baumannii*	28	0	0	100	1	1	100	3	3	100	2	2	100	0	0	100	13	12	92.3	1	1	100	7	7	100	1	1	100	0	0	100	27/28	96.4
*C. braakii*	2	0	0	100	0	0	100	0	0	100	0	0	100	0	0	100	0	0	100	0	0	100	0	0	100	0	0	100	0	0	100	2/2	100
*C. freundii*	1	0	0	100	0	0	100	0	0	100	0	0	100	0	0	100	0	0	100	0	0	100	0	0	100	0	0	100	0	0	100	1/1	100
*E. cloacae*	5	0	0	100	0	0	100	0	0	100	0	0	100	1	1	100	0	0	100	0	0	100	0	0	100	0	0	100	2	2	100	5/5	100
*E. coli*	4	2	2	100	0	0	100	1	1	100	0	0	100	1	1	100	0	0	100	0	0	100	0	0	100	0	0	100	0	0	100	4/4	100
*K. oxytoca*	2	0	0	100	2	2	100	0	0	100	0	0	100	0	0	100	0	0	100	0	0	100	0	0	100	0	0	100	0	0	100	2/2	100
*K. pneumoniae*	63	25	25	100	6	6	86	1	1	100	0	0	100	31	30	97	0	0	100	0	0	100	0	0	100	0	0	100	0	0	100	62/63	98.4
*P. aeruginosa*	12	0	0	100	4	4	100	0	0	100	0	0	100	0	0	100	0	0	100	0	0	100	0	0	100	0	0	100	8	8	100	12/12	100
**Total Concordance**	**117**	**27**	**27**	**100**	**13**	**13**	**100**	**5**	**5**	**100**	**2**	**2**	**100**	**33**	**32**	**96.9**	**13**	**12**	**92.3**	**1**	**1**	**100**	**7**	**7**	**100**	**1**	**1**	**100**	**10**	**10**	**100**	**115/117**	**98.2**

a All tested *C. braakii* and *C.freundii* isolates and 2 tested *E. cloacae* isolates did not show carbapenemase activity and were exclusively used as control strains for species specific marker, see table 2.

b Includes isolates producing KPC-1 (n = 1), KPC-2 (n = 18) and KPC-3 (n = 8).

c Includes isolates producing VIM-1 (n = 7), VIM-2 (n = 4) and VIM-4 (n = 4).

d Includes isolates producing NDM-1 (n = 4) and NDM-2 (n = 1).

e All isolates produce GIM-1.

f Includes isolates producing OXA-48 (n = 31), OXA-162 (n = 1) and OXA-181 (n = 1); OXA-181 (in *K. pneumoniae*) was not detected by the microarray.

g All isolates produce OXA-23.

h All isolates produce OXA-58.

i All isolates produce OXA-72.t

j Isolate produces OXA-51.

k Includes isolates producing IMP-2 (n = 1), IMP-4 (n = 1), IMP-7 (n = 1), IMP-8 (n = 1), IMP-13 (n = 1), IMP-14 (n = 1), IMP-15 (n = 1), IMP-16 (n = 1), IMP-31 (n = 1) and IMP-unknown (n = 1).

l **R**eference: Genotyping results of the carbapenemases genes were obtained from the National Reference Laboratory for Multidrug-Resistant Gram-Negative Bacteria, Bochum (n = 94), from University Medical Center of Jena and the Robert Koch-Institute, Wernigerode (n = 14). Nine isolates were bought from established strain collections (Institute Pasteur, German Collection of Microorganism and Cell Cultures).

m **M**icroarray-results were obtained using carbapenemase-array designed in this study.

All 82 oligonucleotide primer-probe sets were synthesized by Metabion (Martinsried, Germany) and spotted and manufactured to ArrayStrip microarrays as previously described [39]. Biotinylated oligonucleotides with an artificial sequence and spotting buffer were spotted as a staining and negative control, respectively.

### Multiplex labeling and linear DNA amplification

**Figure 1:**
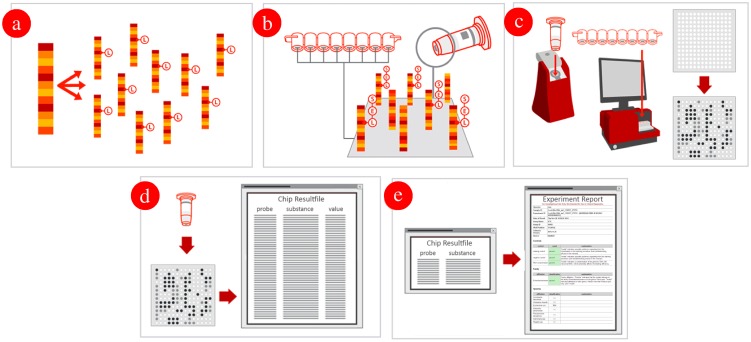
Linear multiplex DNA amplification, labeling and hybridization with the ArrayStrips. (**a**) Linear Multiplex Amplification starting from clonal RNA-free genomic DNA. Extracted DNA is internally labeled with biotin (Label [L]) and amplified in a linear multiplex PCR reaction; (**b**) Hybridization: the internally biotin labeled, single-stranded DNA product hybridizes specifically under stringent conditions to the corresponding probes. The resulting duplex is detected using a horse-radish peroxidase (Enzyme [E]) – streptavidin conjugate, which causes the dye to precipitate ([S]). (**c**) Detection: the ArrayMate Reader (or ArrayTube Reader ATR 03) enables the visualization and subsequently automated analysis of the array image. The presence of a dark precipitated spot indicates successful hybridization; (**d**) Analysis: the assay specific software analysis script coming with the ArrayMate Reader (or ArrayTube Reader ATR 03), measures the signal intensity of each probe and determines which genes/alleles are present in the sample by means of an assay specific algorithm. (**e**) Genotype analysis: a software plugin coming with the ArrayMate Reader (or ArrayTube Reader ATR 03) analyzed the raw data automatically, finally a report is provided on the detected carbapenemases genes.

For the repeated multiplex primer extension amplification, a set of 75 primers (60 primers for antimicrobial resistance targets and 15 species-specific primers) was used (Table S2). Each primer is located directly adjacent to and downstream of his corresponding covalently immobilized oligonucleotide detection probe (Tables S3 and S4). The absolute number of probes and primers does not need to be identical since every primer or probe can be specified by one or more counterparts. The internal labeling of the synthesized single stranded DNA resulted from the primer elongation of previously hybridized primers to the target genomic DNA by using dUTP linked biotin as dideoxynucleotidetriphosphate to be incorporated during synthesis. This procedure allowed site-specific internal labeling of the corresponding target region (Figure 1a). Using the HybPlus Kit (Alere Technologies), at least 0.5 µg genomic DNA was labeled according to manufacturer's instructions. The optimized PCR protocol included 5 min of initial denaturation at 96°C, followed by 50 cycles with 20 s of annealing at 50°C, 40 s of elongation at 72°C, and 60 s of denaturation at 96°C. This reaction results in a multitude of specifically amplified, single-stranded, biotin-labeled DNA molecules for subsequent hybridization and detection using the DNA microarray.

### Hybridization, staining and data analysis

For hybridization procedures, the HybPlus Kit (Alere Technologies) was used according to manufacturer's instructions with an adapted hybridization protocol. This included hybridization buffer C1, washing buffer C2, peroxidase-streptavidin conjugate C3, conjugation buffer C4, washing buffer C5 and peroxidase substrate D1. Carbapenemase ArrayStrips were placed in a thermo mixer with an Alere ArrayStrip adapter (Order no. 1808–1081 and Order no. 1808–0506, Quantifoil Instruments, Jena, Germany) and subsequently washed with 200 µl of deionized water at 50°C/550 rpm for 5 min and with 100 µl hybridization buffer C1 at 50°C/550 rpm for 5 min. All liquids were always completely removed using a soft plastic pipette (i.e. #612–2856; BRANDT, Wertheim, Germany) to avoid any scratching of the chip surface. In a separate tube, 10 µl of previously labeled, single-stranded DNA were dissolved in 90 µl hybridization buffer C1. The hybridization was carried out at 50°C and 550 rpm for 1 h. After hybridization, the ArrayStrips were washed two times using 200 µl washing buffer C2 at 45°C for 5 min, shaking at 550 rpm. Peroxidase-streptavidin conjugate C3 was diluted 1∶100 in buffer C4. A total of 100 µl of this mixture were added to each well of the ArrayStrip, and subsequently incubated at 30°C and 550 rpm for 10 min. Afterwards, two washing steps with C5 washing buffer were carried out at 550 rpm with 200 µl at 30°C for 5 min. The visualization was achieved by adding 100 µl of staining substrate D1 to the ArrayStrips, and signals were detected using the ArrayMate device (Alere Technologies) (Figure 1b–c). This final protocol was optimized by systematic testing of different hybridization temperatures (45°C–58°C) and washing temperatures (45°C–58°C).

Hybridization signals were processed using the IconoClust software, version 3.2r1 (Figure 1d). All spots were recognized and subsequently normalized automatically by the software according to the equation *NI* = *1*- (*M*/*BG*) where *NI* is the normalized intensity, *M* the average intensity of the automatically recognized spot, and *BG* the intensity of the local background [39]–[41]. The output range of the signals was between 0 and 1, with 0 being negative and 1 being the maximum possible signal value. Calculated data were combined in a grey-value-table for all probes. Finally, a HTML-report is provided giving information on the presence of antimicrobial resistance genes and the affiliation to one of the more common species (Figure 1e).

### PCR for alternative detection of different beta-lactamases

PCRs specifying consensus regions for additional carbapenemases, ESBLs and NSBLs were conducted as a supplementary method to the microarray-based assay. All PCRs were designed for use of the specific reverse primer from the multiplex primer extension amplification of the labeling procedure and a newly designed forward primer specific for the particular beta-lactamases gene (Table 3). Additionally, a *bla*OXA-23 PCR was performed to control for the *OXA-23* negative strain (CARB048, Table S1), which was tested positive elsewhere ([42]; OXA-23-likeF: 5′-GATCGGATTGGAGAACCAGA-3′, OXA-23-likeR: 5′-ATTTCTGACCGCATTTCCAT-3′; 501 bp). For each single PCR reaction, 5 µl QIAGEN Multiplex PCR Master Mix (Cat.No. 206143; QIAGEN, Hilden, Germany), 0.05 µl of each primer (100 pmol), 2.9 µl molecular biology grade water and 2 µl genomic RNA-free DNA (app. 200 ng) were used. The PCR protocol for all targets included 4 min of initial denaturation at 95°C, followed by 35 cycles with 30 s of denaturation at 95°C, 30 s of annealing at 50°C, and 60 s of elongation at 72°C. Finally an elongation at 72°C was conducted for 10 min. Amplicons were separated using a 1.5% agarose gel at 130 V and analyzed by a gel documentation system (Bio-Rad Laboratories GmbH, Munich, Germany).

**Table 3:** Additional lactamases detected by the microarray in comparison to conducted control PCRs.

	Primer forward	Sequence	Primer reverse	Sequence	lengths (bp)	Reference	No. of Isolates	No. of Isolates	% Concordance
							M^a^	PCR^b^	
blaOXA-1	prim_oxa1_20_fwd	5′-TTCTGTTGTTTGGGTTTCGC-3′	prim_oxa1_20	5′-ACGCAGGAATTGAATTTGTTC-3′	190	this study	16	16	100
blaOXA-2	prim_oxa2_10_fwd	5′-GTTAATGGCAATCCGAATCTT-3′	prim_oxa2_10	5′-GGATCGTGCCATGTTGG-3′	176	this study	4	4	100
blaOXA-9	prim_oxa9_10_fwd	5′-TGTGTCTCCGTGCTCGTCTT-3′	prim_oxa9_10	5′-CCGATCAACTCCCAGACG-3′	207	this study	33	33	100
blaOXA-10	prim_oxa7_10_fwd	5′-TGGAACAAAGAGTTCTCTGCC-3′	prim_oxa7_10	5′-GGCTTTCCGTCCCATTTG-3′	207	this study	3	3	100
blaOXA-51	lb_oxa_654_fwd	5′-AGCCTGCTCACCTTATATAGTGACT-3′	lb_oxa_654	5′-TTGAAGGTCGAAGCAGGTA-3′	201	this study	25	25	100
blaOXA-58	lb_oxa_657_fwd	5′-GCCAATGCACTAATTGGTTTAGA-3′	lb_oxa_657	5′-GCAATTCACTTTGCATTAAGCT-3′	193	this study	2	2^c^	100
blaCTX-M1/15	prim_ctxM1_10_fwd	5′-AGGCGTTTTGACAGACTATTCA-3′	prim_ctxM1_10	5′-CCGTTTGCGCATACAGC-3′	152	this study	17	17	100
blaVIM	DG_VIM-cons_fwd	5′-GATGGYGTTTGGTCGCATATCKCAAC-3′	DG_VIM-cons_rev	5′-CGAATGCGCAGCACCRGGATAGAA-3′	390	this study	1	1^d^	100
						**Total concordance**	**101**	**101**	**100**

a Results were obtained using the microarray-based carbapenemases array, designed in this study.

b Results were obtained by PCR designed in this study.

c One *bla*OXA-58 was detected in an *OXA-23* negative strain tested by microarray, which was originally tested as an *OXA-23* positive strain by the national reference center.

d *Bla*VIM was detected in addition to an IMP carbapenemase gene.

## Results

The designed microarray included probes to analyze genes encoding clinically important carbapenemases (i.e. *bla*VIM, *bla*KPC, *bla*NDM) as well as narrow spectrum beta lactamases (i.e. *bla*OXA-1, *bla*OXA-7) and extended spectrum beta-lactamases (*bla*CTX-M1, *bla*CTX-M15). Additionally, we included 15 species specific targets for *E. coli, A. baumannii, K. pneumoniae, P. aeruginosa* and *C. freundii/braakii*. Verification and testing of the array was completed using different carbapenemase positive Gram-negative bacteria derived from human sources (raw data provided in Table S6).

### Phenotyping and genotyping by external reference laboratories

The phenotypic antimicrobial resistance determination of the isolates was done at the university hospital medical centers of Dresden (UMC Dresden) and Jena (UMC Jena) during routine procedures either by the VITEK 2 system or by agar diffusion assays (Table S1). Previously characterized strains that were received from culture collections (DSMZ, Institut Pasteur) were not re-tested phenotypically. The genotypic analysis of carbapenemase producing isolates was done at the German National Reference Laboratory for Multidrug-Resistance Gram-negative Bacteria at Bochum University by order of the UMC Dresden. Isolates from the UMC Jena were genotypically identified at the Robert Koch-Institute (Wernigerode, Germany) and all other isolates were bought as reference strains from international culture collections (DSMZ and Institut Pasteur). Both the phenotype and the genotype were compared with the microarray-based assay described in this study. A detailed overview of the genotyping results from the reference centers is given in the Table S1.

### Verification of the assay

A set of 112 strains that were otherwise confirmed to produce carbapenemases was used to verify the current DNA-based microarray for carbapenemase detection. Additionally, five strains sensitive to carbapenem compounds [*C. freundii* (n = 1), *C. braakii* (n = 2), *E. cloacae* (n = 2)] were additionally used to verify only species specific markers. Carbapenemase genes *bla*GIM-1 (n = 2), *bla*IMP-2 (n = 2), *bla*IMP-4 (n = 1), *bla*IMP-7 (n = 1), *bla*IMP-8 (n = 1), *bla*IMP-13 (n = 1), *bla*IMP-14 (n = 1), *bla*IMP-15 (n = 1), *bla*IMP-16 (n = 1), *bla*IMP-31 (n = 1), *bla*KPC-2 (n = 19), *bla*KPC-3 (n = 8), *bla*NDM-1 (n = 4), *bla*NDM-2 (n = 1), *bla*OXA-48-group [blaOXA-48 (n = 31), *bla*OXA-162 (n = 1), *bla*OXA-181 (n = 1)], *bla*OXA-23 (n = 13), *bla*OXA-51 (n = 1), *bla*OXA-58 (n = 1), *bla*OXA-40-group [*bla*OXA-72 (n = 7)], *bla*VIM-1 (n = 5), *bla*VIM-2 (n = 4) and *bla*VIM-4 (n = 4) were identified by PCR, performed at NRL, DSMZ or Institut Pasteur (Table S1). The microarray-based assay gave concordant results for 115 of 117 strains harboring genes for GIM-1, IMP-2, IMP-4, IMP-7, IMP-8, IMP-13, IMP-14, IMP-15, IMP-16, IMP-31, KPC-2, KPC-3, NDM-1, NDM-2, OXA-162, OXA-23, OXA-48, OXA-51, OXA-58, OXA-72, VIM-1, VIM-2 and VIM-4. In our study, the overall concordance between the microarray-based assay and the reference methods (genotyping by NRL) was 98.2% and the gene-specific concordance was 100% for *bla*KPC, *bla*VIM, *bla*NDM, *bla*GIM, *bla*IMP, *bla*OXA-51, *bla*OXA-58 and *bla*OXA-40-group (Table 2). The gene-specific concordance was 97% for *bla*OXA-48-group and 99% for *bla*OXA-23 (Table 2). For one isolate producing *OXA-23* (CARB048, Table S1) and one isolate producing *OXA-181* (CARB097, Table S1), the resistance genes were not detected by the microarray-based assay. For the former isolate, which was originally analyzed as an OXA-23 producer, the gene encoding the carbapenemase OXA-58 was determined. This result was confirmed by the *bla*OXA-58 PCR (Table 3). The carbapenemase resistance gene *bla*OXA-181 belonging to the OXA-48-group was not identified, due to 7 mismatches within the *bla*OXA-48 consensus probe on the microarray. All four carbapenemase negative strains yielded correct negative results. An additional *bla*VIM gene was found in a strain (NRZ-00648, Table S1) harboring an *IMP* carbapenemases gene. This result was confirmed by the *VIM-s*pecific control PCR (Table 3).

The overall concordance rate for species identification was 97.4% (114/117). The species specific concordance of the assay for *E. coli*, *A. baumannii, C. braakii/freundii*, *E. cloacae* and *P. aeruginosa* was 100% (Table 4). Of all analyzed strains only three were not identified in concordance with the phenotypical analysis. These included one *K. pneumoniae* strain (in which the species-specific marker *khe* was not detected) and two *K. oxytoca* strains (for which no species-specific marker was present on the microarray). The species-specific concordance for *K. pneumoniae* was 98.4% (62/63) and for *K. oxytoca* 0% (0/2). The selected species marker *pld, efp* and *basC* identified *A. baumannii* correctly, and yielded negative results for all other species tested on the array. For *E. coli*, *gad* was a reliable species-specific marker; all other tested species were negative. The probe for *ecfX* was positive only for *P. aeruginosa* and negative for all other tested species in this study. For *Enterobacter spec*. the probes *dnaE* and *ihfA* identified this genus, if the probe for *gad* was negative. The probe for *ihfA* was positive for all *Enterobacteriaceae* (*E. coli*, *K. oxytoca*, *K. pneumoniae* and *Enterobacter spec*.) but negative for species belonging to *Pseudomonadales* (*A. baumannii, P. aeruginosa*). A reliable marker for *C. freundii* and *C. braakii* was the probe for *cfa*, other tested *Citrobacter* species (*C. gilleni*, *C. koseri*) were negative (data not shown).

**Table 4:** Species genotyping results of the **M**icroarray-based assay in comparison to the **R**eference method (phenotypic results were obtained from University Medical Center of Dresden, University Medical Center of Jena, German Collection of Microorganisms and Cell Cultures, Institut Pasteur and Friedrich-Loeffler-Institute).

Species	No. of isolates	*A. baumanii* (*basC, efp, pld*)		% Concordance	*C. braakii/freundii (cfa*)^a^		% Concordance	*Enterobacter spec. (dnaE, ihfA)* b		% Concordance	*E. coli* (*gad, dnaE, ihfA*)		% Concordance	K. oxytoca^c^		% Concordance	K. pneumonia (*khe*)		% Concordance	P. aeruginosa (*ecfX*)		% Concordance	No. of strains that agree/total no. of strains	% Concordance of all strains
		R^d^	M^e^		R	M		R	M		R	M		R	M		R	M		R	M			
*A. baumannii*	28	28	28	100	0	0	100	0	0	100	0	0	100	0	0	100	0	0	100	0	0	100	28/28	100
*C. braakii/freundii*	3	0	0	100	3	3	100	0	0	100	0	0	100	0	0	100	0	0	100	0	0	100	2/2	100
*Enterobacter spec.*	5	0	0	100	0	0	100	5	5	100	0	0	100	0	0	100	0	0	100	0	0	100	5/5	100
*E. coli*	4	0	0	100	0	0	100	0	0	100	4	4	100	0	0	100	0	0	100	0	0	100	4/4	100
*K. oxytoca*	2	0	0	100	0	0	100	0	0	100	0	0	100	2	0	0	0	0	100	0	0	100	0/2	0
*K. pneumoniae*	63	0	0	100	0	0	100	0	0	100	0	0	100	0	0	100	63	62	98.4	0	0	100	62/63	98.4
*P. aeruginosa*	12	0	0	100	0	0	100	0	0	100	0	0	100	0	0	100	0	0	100	12	12	100	12/12	100
**Total Concordance**	**117**	**27**	**27**	**100**	**3**	**3**	**100**	**5**	**5**	**100**	**5**	**5**	**100**	**2**	**0**	**0**	**63**	**62**	**98.4**	**5**	**5**	**100**	**114/117**	**97.4**

a Includes one isolate *C. freundii* and two isolates *C. braakii*.

b All isolates were *Enterobacter cloacae*.

c All tested *K.oxytoca* isolates were not detected by the microarray, due to missing species-specific marker.

d **R**efence: Phenotypic results were obtained from University Medical Center of Dresden (n = 89), University Medical Center of Jena (n = 13), German Collection of Microorganisms and Cell Cultures (n = 3), Institut Pasteur (n = 1), Friedrich-Loeffler-Institute (n = 2).

e **M**icroarray-results were obtained with the microarray-based carbapenemases array, designed in this study.

Additionally to the carbapenemase specific oligonucleotides, also sequences for different beta-lactamases (*bla*OXA-1, *bla*OXA-2, *bla*OXA-7, *bla*OXA-9, *bla*OXA-10 and *bla*OXA-60) and one marker for an extended spectrum beta-lactamase (*bla*CTX-M1/M15) were integrated. Using the microarray-based assay we could determine within the set of isolates additional beta lactamase genes [*OXA-1* (n = 16), *OXA-2* (n = 4), *OXA-9* (n = 33), *OXA-10* (n = 3), *CTX-M* (n = 17), *OXA-51* (n = 25), *OXA-58* (n = 2), *VIM* (n = 1)] for which these isolates have not previously been tested. For all these beta lactamase genes control PCRs were performed and the overall concordance rate between microarray and PCR method was 100% (Table 3).

## Discussion

The carbapenemase encoding genes analyzed by the described microarray-based assay comprise the majority of the carbapenemases found in *Enterobacteriaceae* and *Pseudomodales* [43]. Thus, the molecular assay offers convenient, fast, economic, parallel and accurate molecular detection of carbapenemase genes for microbiological laboratories. The overall concordance of 98.2% (115/117) is comparable to other commercially available molecular assays for which concordances of 97.6% [44] and 97% [45] have been reported.

The microarray-based assay found a 100% concordance for all carbapenemase genes except of one *bla*OXA-23 and one *bla*OXA-48-like gene. Also, all species within the set of isolates were correctly identified except of one *K. pneumoniae* and two *K. oxytoca* strains. The described test found an additional *VIM* gene in a reference strain that was tested positive for *bla*IMP-8, which was also confirmed by PCR (Table 3). In this case it was not clear whether the phenotypic resistance can be attributed to *IMP-8* or *VIM* or to both. Furthermore, the *OXA-51* carbapenemase gene was additionally found in different isolates (n = 25), in addition to the genes which were identified by the reference centers (Table S1). The large number of additionally detected *OXA-51* genes can be explained by the consensus probe designed for all enzymes of the *OXA-51*-group, which will also be positive for all other subtypes of this group [46]. Therefore, the described assay cannot distinguish between allelic members within this group, but for a clinically relevant report, a differentiation is not necessary as all enzymes from this group can hydrolyze carbapenem compounds. An *OXA-181* gene in one *K. pneumoniae* isolate was not detected because that gene was not yet included by the array. Compared to other OXA-48-like genes, it shows multiple mismatches within the probe binding region. For this gene a new probe will be designed and included in the next microarray design. One isolate was tested positive for *bla*OXA-23 by the NRL but this gene was neither found using the microarray nor a specific PCR for *bla*OXA-23 [42]. A repeated susceptibility test using the VITEK 2 system showed that this strain was still resistant against carbapenemases ruling out a possible plasmid loss. We analyzed this strain again using the microarray-based method and found two other genes encoding carbapenem hydrolyzing enzymes, *OXA-51* and *OXA-58*. The presence of both genes was confirmed by PCR. This strain was the only one revealing non-concordant results between the microarray-based assay and control methods. A possible explanation is that the original isolate was in fact a mixed culture, and that different clones have been picked and further processed by the respective laboratories. The included probe for *bla*KHM could not be verified because no KHM-positive reference strain was available. *Bla*KHM is very rare worldwide and it was first described in Japan [47]. However, given its conserved sequence and the highly similar biophysical characteristics of all probes and primers, a correct detection of *bla*KHM can be expected. All additional beta-lactamases found by the microarray were confirmed by alternative PCR methods (Table 3). These results highlight the ability to detect and identify multiplex amplification products of carbapenemase, NSBL and ESBL genes.

The integrated markers for *K. pneumoniae*, *C. freundii*/*braakii*, *E. coli*, *P. aeruginosa*, and *A. baumannii* correctly identified these species. The specific markers for *Enterobacter* spec. identified this genus correctly. Only one *K. pneumoniae* strain was not identified by the probe specific for the *khe* gene [48]. This can be explained by the diversity of *khe* alleles among *K. pneumoniae* strains. Yin-Ching and colleagues found three different sub-types of *HindIII* banding patterns obtained from just 15 isolates. While probe and primer have both been placed in regions that were conserved among 45 published *khe* sequences (Table S4), it still could have been the case that sequence variations affecting probe and/or primer binding sites might have caused a negative result in that particular isolate. Nevertheless, the next array design will include additional *khe* probe-primer combinations for other consensus regions of this gene. Within the tested panel, two *K. oxytoca* isolates harboring *bla*VIM were analyzed. The resistance genes for carbapenems were reliably found, but no species specific marker was yet included for this species. In *K. oxytoca* only the family-specific marker *ihfA* was detected and the next microarray design might include an additional specific marker for *K. oxytoca*. Species and genus marker were only tested with the most common of the clinically relevant species. Other genera and species are not yet covered by the array and were not yet experimentally evaluated, assuming that a phenotypic identification of isolates will be available anyway from a diagnostic workflow. However, the assay can easily be expanded to include additional species markers if the necessity arises. Information about possible cross reactions of other species with the included species marker is shown in the Table S5.

The newly developed assay provides an accurate and convenient tool to identify and discriminate the most clinical relevant carbapenemases. Comparable commercially available approaches for detection of carbapenemases are single or multiplex PCRs for known carbapenemases [27], [41]–[45]. These assays are time consuming, labor intensive and require expert knowledge of carbapenemases epidemiology. Detailed PCR analysis of carbapenem-resistant isolates is normally focused only on prevalent carbapenemase genes ignoring other beta-lactamases. Thereby, information about genes encoding other carbapenemases and of clinically important NSBLs and ESBLs is not provided. Besides, it might in some cases also be of relevance to genotypically identify ESBLs, especially with regard to the effect of a simultaneous presence of OXA-1 and CTX-M15 on beta-lactam plus sulbactam/tazobactam combinations [46], [47]. These relevant disadvantages can be resolved by an array-based approach.

The main advantage of the method described herein is a simultaneous analysis of multiple carbapenemases, extended spectrum beta-lactamases (ESBL) and narrow spectrum beta-lactamases (NSBL) in combination with the species detection directly from clonal culture material.

Further advantages of the assay described herein are (a) a controlled and permanently reviewed in-house database for all carbapenemase, ESBL and NSBL sequences that are covered by the assay, (b) the fully automatic analysis by an integrated software tool, which cannot be manipulated by the user, (c) a 100% standardization that allows exchange and comparison of results between laboratories worldwide and (d) a possible combination with fast and economic DNA isolation methods such as the described heat lysis protocol which help to reduce overall costs.

A superficially similar approach to the system described in this study was developed by Check-Points and published first by Naas and colleagues in 2011 [39]. However, Check-Points array uses a different methodology called multiplex ligation detection reaction (LDR), where two target-specific probes are ligated to each other when hybridized to a specific target sequence. DNA molecules generated in this process are then subsequently amplified by PCR with a single pair of primers. The PCR products are hybridized to a low-density DNA microarray. Positive hybridization is detected using biotin labels introduced with one of the PCR primers. Advantages of this method include the ability to recognize SNPs (single nucleotide polymorphism) and a possible use on samples with low DNA concentrations, i.e., DNA isolated directly from a patient sample. While it can be helpful to detect certain resistance genes directly from this kind of samples, direct typing is nevertheless not feasible because many patient samples hold multiple species and strains of Gram-negative bacteria simultaneously. Due to the exponential amplification during PCR, the assay becomes more susceptible to contamination. Additionally, the Check-Points assay is complex and a target update would require both the inclusion of new target-specific probes for LDR and the introduction of additional probes for detection on the microarray. Given the permanently increasing number of relevant target genes and of available sequences, these difficulties of up-dating and expanding this assay might pose a limitation for this concept.

The relative costs of commercially available assays are reviewed by Dortet and colleagues in 2014 [49] providing a schematic classification of overall effective prices for various test systems. If costs are calculated only for the used reagents, the microarrays and the ArrayMate reader, the overall price of the assay described in this study would then be in a range between the CarbaNP- and the PCR tests. With regard to rapidity, our assay requires, when starting from a clonal culture, less than 8 hours with an overall hands-on time of approximately 2 hours. Furthermore, the assay is able to identify mixed or contaminated cultures due to the inclusion of species-specific markers. Due to the multiplexing design more than one carbapenemase could be identified at the same time. The results from the assay are analyzed completely automated using the ArrayMate device and a final HTML-report is provided giving information on the antimicrobial resistance genes of the species and the species itself. Furthermore, up to 96 samples can be analyzed simultaneously so that the described test is also suitable for laboratories with a high sample throughput. As with all molecular tools, this assay only identifies known genes, and cannot detect novel genes or genes not yet included. Upgrading the assay for additional targets, such as discussed above for a modified probe for *khe* and/or probes to identify *K. oxytoca*, is simple and can be done promptly once the requirement arises. At a later date, we aim to transfer the described assay into a completely automated point-of-care (POC) device.

## Supporting Information

Table S1 **Detailed comparison between phenotype and genotype of the used isolates.**
(XLSX)

Table S2 **Sequences of the used set of labeling primers for the linear extension PCR.**
(XLSX)

Table S3 **Probes spotted on the microarray.**
(XLSX)

Table S4 **Primer and probe distance and matching within the target sequences.**
(XLSX)

Table S5 **Matching of species specific probes to target sequences (numbers indicate mismatches numbers within the target sequence).**
(XLSX)

Table S6 **Microarray raw data of all performed experiments.**
(XLSX)
