# Correction: Rapid Identification of Carbapenemase Genes in Gram-Negative Bacteria with an Oligonucleotide Microarray-Based Assay

**DOI:** 10.1371/journal.pone.0107079

**Published:** 2014-09-02

**Authors:** The PLOS ONE Staff

## Notice of Republication

This article was republished on August 7, 2014, to replace incorrectly changed characters in the byline, Competing Interests statement, Author Contributions, and Acknowledgments in the online version only. The publisher apologizes for the errors. 
